# The assessment of pressure-volume relationship during exercise stress echocardiography predicts left ventricular remodeling and eccentric hypertrophy in patients with chronic heart failure

**DOI:** 10.1186/s12947-019-0157-z

**Published:** 2019-04-06

**Authors:** Iacopo Fabiani, Nicola Riccardo Pugliese, Claudia Santini, Mario Miccoli, Andreina D’Agostino, Ilaria Rovai, Matteo Mazzola, Roberto Pedrinelli, Frank Lloyd Dini

**Affiliations:** 1 Cardiac, Thoracic and Vascular Department, University of Pisa, Pisa, Italy; 2 Department of Surgical, Medical, Molecular and Critical Care Pathology, Fresno, USA; 3 Department of Clinical and Experimental Medicine, University of Pisa, Pisa, Italy

**Keywords:** Stress echocardiography, Heart failure, Left ventricular remodeling

## Abstract

**Background:**

The contractile response of patients with heart failure (HF) may be assessed by exercise stress echocardiography (ESE)-derived indexes. We sought to test whether ESE parameters are useful to identify the risk of adverse left ventricular (LV) remodeling in patients with chronic HF and reduced or mildly reduced LV ejection fraction (EF).

**Methods:**

We enrolled 155 stabilized patients (age: 62 ± 11 years, 17% female, coronary artery disease 47%) with chronic HF, LV EF ≤50% and LV end-diastolic volume index > 75 ml/m^2^. All patients underwent a symptom-limited graded bicycle semi-supine ESE, with evaluation of peak stress LV EF, end-systolic pressure-volume relation (ESPVR, i.e. LV elastance) and cardiac power output to LV mass (CPOM). A complete echocardiographic study was performed at baseline and after 6 ± 3 months. Adverse LV remodeling was defined as the association of eccentric LV hypertrophy (LV mass: ≥115 g/m^2^ for male and ≥ 95 g/m^2^ for women, and relative wall thickness < 0.32) with an increase in LV end-systolic volume index ≥10% at six months.

**Results:**

Adverse LV remodeling was detected in 34 (22%) patients. After adjustment for clinical, biochemical and echocardiographic data, peak ESPVR resulted in the most powerful independent predictor of adverse LV remodeling (OR: 12.5 [95% CI 4.5–33]; *p* < 0.0001) followed by ischemic aetiology (OR: 2.64 [95% 1.04–6.73]; *p* = 0.04).

**Conclusion:**

In patients with HF and reduced or mildly reduced EF, a compromised ESE-derived peak ESPVR, that reflects impaired LV contractility, resulted to be the most powerful predictor of adverse LV remodeling.

## Introduction

In patients with left ventricular (LV) dysfunction and reduced ejection fraction (LVEF), transition to heart failure (HF) is often accompanied by progressive LV dilation and changes in ventricular architecture (the so-called LV remodeling) and decline in wall thickness-to-cavity radius, which is referred to as relative wall thickness (RWT), resulting in eccentric LV hypertrophy (LVH) [1]. LV remodeling may occur after myocardial infarction, hemodynamic overload or primary myocardial disease [2–4]. Although the etiologies of these disorders are different, they share several pathways that ultimately lead to similar changes in LV size, shape and function [5]. The ensuing development of pathological LVH and LV remodeling may ultimately be associated with a depression of LV performance and the intrinsic contractile state of the myocardium [6].

Stress echocardiography, including dobutamine stress and exercise stress echocardiography (ESE), may endow with additional insights for the evaluation of LV performance and the prediction of changes in LV volumes [7]. Evaluating global myocardial function by stress echocardiography is crucial to distinguish between adaptive or maladaptive LV remodeling in patients with LV systolic dysfunction. The identification of viable tissue relies on the identification of an enhanced contractile response in segments of apparently non-contractile myocardium, usually supplied by stenosed coronary arteries. On the contrary, the assessment of the global pumping capacity during dobutamine challenge or exercise testing is essential to examine the overall ability of the myocardium to comply with the hemodynamic and metabolic needs of the whole body [8, 9]. A number of stress echo studies have been conducted to evaluate the global contractile response of dysfunctional myocardium in patients with HF using different parameters, including changes in LVEF, changes in wall motion score index, changes in end-systolic volume (ESV), cardiac power output-to-mass (CPOM) and the end-systolic pressure-volume relation (ESPVR; i.e. LV elastance) [10–14]. In the present study, we sought to investigate whether the assessment of LV contractile performance during ESE testing may be useful to predict the risk of adverse LV remodeling in patients with HF and reduced or mildly reduced LVEF.

## Methods

### Patients

This study included a total of 155 outpatients with HF enrolled between 2013 and 2016 at the Cardiac, Thoracic and Vascular Department, University of Pisa, Pisa, Italy. The inclusion criteria comprised LVEF ≤50% and LV end-diastolic volume index (EDVi) > 75 ml/m^2^. The exclusion criteria were: HF secondary to degenerative valvular heart disease, peripheral artery disease limiting the capability of performing exercise stress test and reduced exercise tolerance attributable to myocardial ischemia or advanced HF (New York Heart Association, NYHA, functional class > III). Of the 168 patients initially selected for the study, 13 were subsequently excluded: six owing to inability to perform the exercise, six due to poor image quality during the exercise test and one due to the occurrence of a second-degree atrioventricular block during exercise. The study patients were clinically stable and under oral treatment. Beta blockers were withheld at least 48 h before the test.

### Echocardiography

All patients underwent transthoracic two-dimensional and Doppler echocardiographic examination at baseline, during bicycle semi-supine exercise and after follow-up with commercial equipment using 2nd-harmonic imaging and a 3.5-MHz transducer: Acuson Sequoia C256 (Siemens, Mountain View, California) and iE33 X-matrix(Philips, Einthoven, The Netherlands).

Before exercise and during follow-up, a complete echocardiographic and Doppler examination was performed with the subject in the left lateral supine position. The standard parasternal long-axis view as well as the three standard apical four-chamber, two-chamber and long-axis views were acquired optimising gain setting, sector angle and depth. LV end-diastolic volume (EDV) and ESV were calculated according to the biplane Simpson’s rule. LV ESV index (ESVi) and EDVi were assessed by dividing LV volumes for body surface area. The LV mass index was determined by using the M-mode method according to the Recommendations of the American Society of Echocardiography and the European Association of Cardiovascular Imaging [15]. The LV outflow tract anteroposterior diameter was measured in the parasternal long-axis view and the LV outflow tract area was calculated as πr^2^ (cm^2^). The LV stroke distance (cm) was measured tracing the outer edge of the densest (or brightest) portion of the spectral aortic tracing recorded from the apical 5-chamber view, with the PW Doppler sample volume positioned about 5 mm proximal to the aortic valve [16]. Doppler tissue imaging longitudinal velocities were recorded with the sample volume placed at the junction between the septal and lateral LV wall and the mitral annulus in the 4-chamber view and peak early myocardial wave (e’) velocities were measured. The ratio of mitral E peak velocity and averaged e’ velocity (E/e’) was calculated [17]. Patients with a more than mild mitral regurgitation were identified according to the vena contracta method.

### Patterns of left ventricular geometry

Allocation of patients into categories of LV geometry was made by reference to the Guidelines for Chamber Quantification: where LVH was assigned for values ≥115 g/m^2^ for male and ≥ 95 g/m^2^ for women [15]. A cut-off value for RWT of 0.32 was used to categorize geometric patterns: patients with normal LV mass can have either normal pattern (normal LV mass with RWT ≥ 0.32) or eccentric non-hypertrophied pattern (RWT < 0.32), while patients with increased LV mass can have either non-eccentric (RWT ≥ 0.32) or eccentric (RWT < 0.32) hypertrophy [18].

### Definition of adverse LV remodeling

Adverse LV remodeling was defined as the association of eccentric LVH and an increase of LV ESVi [(follow-up LV ESVi – baseline LV ESVi)/baseline LV ESVi] ≥10% [19]. These values were determined via echocardiography during the follow-up period.

### Exercise echocardiography

Symptom-limited graded bicycle semi-supine exercise was performed at an initial workload of 20 watts; afterwords, the workload was increased stepwise by 10 watts every two minutes. A 12-lead electrocardiogram and blood pressure (BP) determination were performed at baseline and during the exercise.

At baseline and during each exercise level, Doppler-derived cardiac output at LV outflow tract, heart rate and arterial systolic and diastolic BP (by cuff sphygmomanometer) were measured. Mean BP (MBP) was estimated as follows: diastolic BP + 1/3 (systolic BP – diastolic BP). Stroke volume was calculated as stroke distance times LV outflow tract area and cardiac output (CO) as stroke volume times heart rate.

The LV end-systolic pressure (ESP) was obtained as 0.9 x systolic BP (mmHg). The ESVPR or LV elastance (mmHg/ml/m^2^) was measured as the ratio of the LV ESP to the LV ESV indexed for body surface area [13].

LV cardiac power output (CPO) was calculated as the product of a constant (K_1_ = 2.22 × 10^-3) with CO (l/min) and MBP (mm Hg). CPOM (W/100 g) was obtained by multiplying CPO by 100 divided by LV mass = K × CO (l/min) × MBP (mmHg) × M^− 1^ (g); K = 2.22 × 10^− 1^ [14]. Echocardiographic images were analysed by two independent cardiologists (I.F., N.R.P.), unaware of the identity of the patient. The intra- and inter-observer reproducibility for systolic function parameters at rest and peak exercise in 20 randomly selected patients were good. Concordance between two raters using the Kappa statistic was 0.95 (*p* < 0.0001).

### Statistics

Data are presented as mean ± standard deviation for continuous variables and as percentages for categorical variables. Normality was assessed via the Kolmogorov-Smirnov test.

Continuous variables were compared using paired and independent samples Student t test or Mann-Whitney and Wilcoxon test, as appropriate.

Categorical variables were compared using the Chi-square or Fisher test, as appropriate. Logistic regression was used to explore the determinants of LV diastolic dysfunction. All variables showing a *p* value < 0.1 at univariate analysis were tested in multivariable models. Stepwise procedure was used to build the multivariate models. The Hosmer-Lemeshow test was used to confirm the goodness-of-fit of multivariable models (*p* = 0.9). The probability level was *p* < 0.05 for all the data examined. Data management and analyses were performed using the IBM SPSS Software Package version 17.0.1.

## Results

Baseline data of the overall patients’ population (62 ± 11 years; 27, 17% female; 87, 56% previous HF history) and in patients categorised according to the presence of adverse remodeling at follow-up are summarized in Table 1. Exercise-echo data are presented in Table 2. The aetiology was ischemic in 72 (46%). Fourty-seven (30%) patients were in NYHA class I, 65 (42%) in class II and 43 (28%) were in class III.

**Table 1:** Baseline characteristics of the study population, according to remodeling pattern at follow-up (Mean/Median and 95% confidence intervals, 95% CI)

Variable	Overall population *n* = 155	No remodeling *n* = 121	Remodeling *n* = 34	*p*-value
Age (yrs)	62 [60–64]	62 [59–65]	61 [59–64]	0.1
Male gender (%)	128 (83%)	95 (79%)	33 (97%)	< 0.0001
Body mass index(kg/m^2^)	26.2 [25.7–27.3]	26.1 [25.4–28.7]	26.2 [25.6–27.5]	0.10
Body surface area (m^2^)	1.9 [1.86–1.93]	1.9 [1.8–2.0]	1.9 [1.86–1.95]	0.10
Coronary artery disease (%)	72 (46%)	51 (42%)	21 (62%)	0.10
Diabetes (%)	28 (18%)	22 (18%)	6 (18%)	0.010
Hypertension (%)	63 (41%)	50 (41%)	13 (38%)	0.003
HF history (%)	87 (56%)	64 (53%)	23 (68%)	0.10
CKD^a^ (%)	51 (33%)	33 (27%)	18 (53%)	0.005
NYHA Class I	47 (30%)	43 (35.5%)	4 (12%)	< 0.0001
NYHA Class II	65 (42%)	49 (40.5%)	16 (47%)	0.0001
NYHA Class III	43 (28%)	29 (24%)	14 (41%)	0.01
BNP (pg/mL)	366 [289–428]	289 [216–362]	613 [451–775]	0.0001
ICD baseline (%)	64 (41%)	40 (33%)	24 (71%)	0.30
CRT baseline (%)	17 (11%)	12 (10%)	5 (15%)	0.04
Mitral regurgitation^b^ (%)	51 (33%)	33 (27%)	18 (53%)	0.005
Digoxin	57 (36.7%)	40 (33%)	17 (50%)	0.14
Diuretics	134 (86%)	100 (83%)	34 (100%)	0.18
ACE inhibitors or ARB	147 (91%)	116 (94%)	31 (96%)	0.47
MRI	102 (66%)	74 (61%)	28 (82%)	0.01
Beta-blockers	109 (70%)	75 (62%)	34 (100%)	0.35

Legend: ACE, angiotensin converter enzyme; ARB, angiotensin receptor blockers; BNP, B-type natriuretic peptide; CKD, chronic kidney disease; CRT, cardiac resynchronization therapy; HF, heart failure; ICD, implantable cardioverter defibrillator; MRI: mineralcorticoid receptor inhibitors; NYHA, New York Heart Association

^a^ *Chronic kidney disease was defined as eGFR < 60 mL/min/1.73m* ^*2*^

^b^ *More than mild mitral regurgitation*

**Table 2:** Resting and peak exercise echocardiographic parameters (Mean/Median and 95% confidence intervals, 95% CI)

Variable	Overall population *n* = 155	No remodeling *n* = 121	Remodeling *n* = 34	*p*-value
Rest				
Heart rate (bpm)	77 [75–79]	77 [75–79]	78 [73–83]	0.60
Systolic arterial pressure (mmHg)	123[120–126]	125 [121–129]	115 [109–121]	0.02
Diastolic arterial pressure (mmHg)	76 [74–78]	77 [75–79]	72 [68–75]	0.06
End-diastolic volume (ml)	200 [192–207]	194 [186–203]	221 [203–239]	0.008
End-systolic volume (ml)	140 [133–147]	134 [126–142]	163 [14.9–1783]	0.001
Ejection fraction (%)	31 [30–32]	32 [31–33]	26 [24–28]	0.0001
Cardiac output (l/min)	4.2 [4.0–4.4]	4.2 [4.0–4.4]	4.1 [3.6–4.5]	0.30
E/e’	13 [12–13]	12 [115–13]	16 [14–18]	0.002
Peak exercise				
Heart rate (bpm)	125 [121–128]	127 [123–129.]	117 [108–125]	0.010
Systolic arterial pressure (mmHg)	162 [157–167.1]	167 [161–173]	143 [132–153]	0.002
Diastolic arterial pressure (mmHg)	99 [82–116]	102 [81–124]	86 [78–94]	0.40
End-diastolic volume (ml)	180 [170–189]	170 [161–179]	215 [190–241]	0.0001
End-systolic volume (ml)	117 [109–124]	108 [100–115]	150 [1479–179]	< 0.0001
Ejection fraction (%)	36 [35–38]	37 [36–39]	30 [29–33]	< 0.0001
Cardiac output (l/min)	8.7 [8.2–9.2]	9.1 [8.6–9.6]	7.3 [6.2–8.5]	0.004
Cardiac power output-to-mass (Watt/100 g)	0.84 [0.78–0.91]	0.91 [0.84–0.98]	0.58 [0.45–0.70]	< 0.0001
LV Elastance (mmHg/ml/m^2^)	1.6 [1.5–1.7]	1.7 [1.62–1.87]	1.0 [0.9–1.2]	< 0.0001

Legend: E/e’, ratio of mitral E peak velocity and averaged e’ velocity; LV, left ventricular

**Fig. 1:**
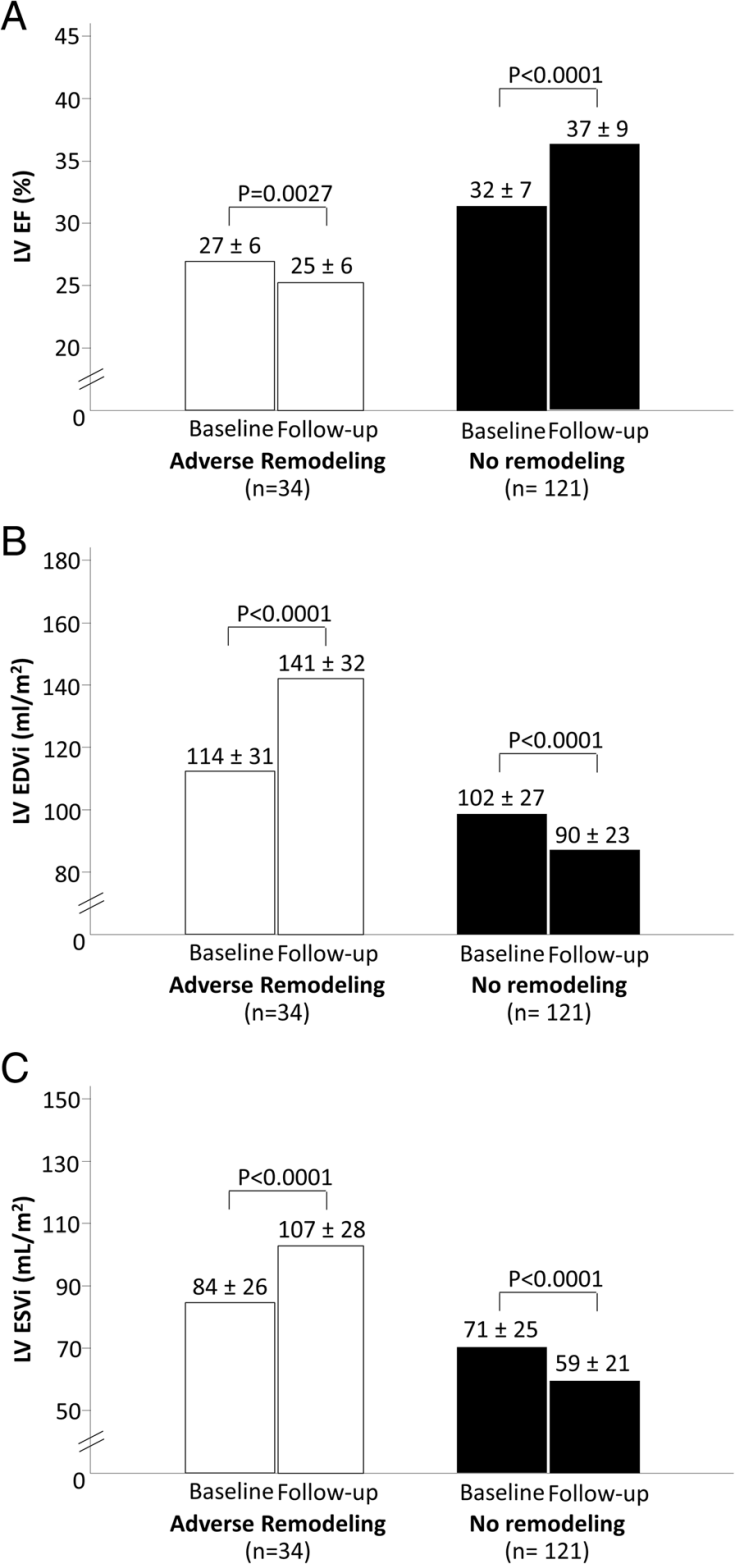
Distribution of **a** left ventricular ejection fraction (LV EF), **b** left ventricular end-diastolic volume index (LV EDVi) and **c** left ventricular end-systolic volume index (LV ESVi) at baseline and follow-up, according to the presence or absence of adverse remodeling

Adverse LV remodeling was reported in 34 (22%) of the study patients at follow-up. Figure 1 shows the baseline and follow-up variations of LVEF, LV EDVi and LV ESVi. After 6 ± 3 months, LVEF increased from 31% (30–32) to 34% (32–35) (*p* < 0.0001), LV ESV decreased from 140 ml (133–147) to 132 ml (123–142) (*p* = 0.01) and LV mass decreased from 146 (140–151) g/m^2^ to 139 (134–145) g/m^2^ (*p* = 0.006). A normal geometric pattern was observed in 8 patients at baseline and in 13 at follow-up, an eccentric non-hypertrophied pattern was reported in 5 at baseline and in 11 at follow-up, a non-eccentric LVH was apparent in 82 at baseline and in 79 at follow-up, while eccentric LVH was present in 60 at baseline and in 52 at follow-up.

Sixty-four of patients exhibited a > 10% reduction in ESVi. Among study patients, 6 underwent percutaneous or surgical correction of functional mitral regurgitation, 3 were submitted to cardiac resynchronization therapy, 2 underwent coronary percutaneous angioplasty and one was revascularized by coronary artery bypass grafting.

Patients with adverse remodeling were prevalently male, presented more advanced NYHA functional class, higher rates of renal dysfunction and moderate to severe mitral regurgitation. Moreover, this group showed higher BNP levels, greater resting and exercise LV volumes, greater LV mass, increased E/e’ratio and more compromised resting and exercise LV EF, peak CPOM and ESPVR.

Univariate and multivariate predictors of adverse LV remodeling are presented in Table 3. By univariate analysis, coronary artery disease, chronic kidney disease, NYHA class, BNP levels, baseline patterns of LV geometry, mitral regurgitation, peak stress LVEF, peak stress LV ESV indexed, peak CPOM and ESPVR were associated to adverse remodeling. In multivariate analysis, peak ESPVR resulted the most powerful independent predictor of adverse LV remodeling (OR: 12.5 [95% CI 4.5–33]; *p* = < 0.0001), followed by ischemic etiology (OR: 2.64 [95% 1.04–6.73]; *p* = 0.04).

**Table 3:** Univariate and multivariate predictors of adverse left ventricular remodeling

Variable	Univariate analysis OR [95% CI]	*p*-value	Multivariate analysis OR [95% CI]	*p*-value
Age (yrs)	0.9 [0.9–1]	0.2		
Gender Male (%)	1.5 [1.3–31]	0.06		
Diabetes Mellitus (%)	0.8 [0.9–1.2]	0.2		
Hypertension (%)	0.9 [0.8–1.1]	0.3		
Coronary artery disease (%)	2.2 [1.01–4.8]	0.001	2.64 [1.04–6.7]	0.04
CKD^a^	1.7 [1.4–4.3]	0.03		
NYHA class (%)	2 [1.2–3.5]	0.05		
BNP (pg/ml)	1.0 [1–1.01]	0.04		
Mitral regurgiation (%)	3 [1.3–6.5]	0.05		
Rest E/e’	1.05 [0.9–1.2]	0.06		
Rest ejection fraction (%)	1.13 [0.7–1.8]	0.59		
Peak ejection fraction (%)	0.50 [0.3–0.8]	0.0059		
Rest end-diastolic volume index (ml/m^2^)	1.0 [0.9–1.2]	0.97		
Peak end-diastolic volume index (ml/m^2^)	1.17 [1.04–1.3]	0.07		
Rest end-systolic volume index (ml/m^2^)	0.98 [0.8–1.2]	0.91		
Peak end-systolic volume index (ml/m^2^)	1.81 [1.2–3.1]	0.05		
Baseline LVMi (g/m^2^)	0.99 [0.97–1.01]	0.81		
Baseline patterns of LV geometry	1.73 [1.3–2.9]	0.04		
Peak cardiac power output-to-mass (Watt/100 g)	0.04 [0.02–0.19]	0.01		
Peak LV elastance (mmHg/ml/m^2^)	8.3 [3.4–25]	< 0.0001	12.5 [4.5–33]	< 0.0001

Legend: BNP, B-type natriuretic peptide; CKD, chronic kidney disease; E/e’, ratio of mitral E peak velocity and averaged e’ velocity; LV, left ventricular; LVMi, left ventricular mass indexed; NYHA, New York Heart Association

^a^Chronic kidney disease was defined as eGFR< 60 mL/min/1.73m^2^

## Discussion

In the present study, conducted in a population of 155 outpatients with HF and reduced or mildly reduced LV EF, we demonstrated that a compromised LV contractility, as reflected by a blunted ESPVR in response to ESE testing, independently predicted adverse LV remodeling as defined by an increased LV mass, RWT < 0.32 and a ≥ 10% increase in LV ESVi.

LV remodeling is usually the result of a progressive process that starts with myocardial damage or excessive LV overload and is very often characterised by the slowly progressive increment of LV volume, increased LV mass and reduced RWT. Since LV overload is initially matched by an adequate growth of cardiac myocytes, the chamber radius is increased, and the wall thickness is increased moderately. To some extent, the initial remodeling may be considered beneficial as the stroke volume may be preserved by augmenting cavity size, but, when the left ventricle further dilates, this adaptive mechanism may progress toward maladaptive remodeling and maladaptive (high-stress) LVH [20–22].

The concept that the occurrence of pathological LVH and LV remodeling in patients with LV overload forms a continuum from a compensatory phase to the stage in which exhaustion and myocardial failure prevail is consistent with the work of Meerson [23]. The first stage reflects the initial myocardial damage due to the imposition of the load. The second stage is described as a phase of relatively stable hyperfunction, in which the increased load is compensated by the increase in myocardial mass. The third stage is that of late deterioration and gradual exhaustion. In the latter stage, a progressive decrease of contractile capacity of the entire ventricle is frequently apparent. It would be, therefore, essential to investigate which are the factors that can predict later cavity enlargement and the decline in wall thickness-to-cavity radius or RWT [24].

The importance of changes in LV shape and volume over time has been addressed in some studies. In 1987, White et al. [25] observed that ESVi measured one to two months after thrombolytic therapy for acute myocardial infarction was a powerful predictor of prognosis, providing additional predictive value to LVEF. A continuous relationship between ESVi and both mortality and hospitalisations for worsening HF has been demonstrated in a similar population. LV EDV measured six months after percutaneous coronary intervention for acute myocardial infarction was clearly associated with worse long-term clinical outcome. In particular, an increase of EDV ≥ 20% resulted to be an independent predictor of survival [26].

In an attempt to provide a comprehensive classification, a new categorization capable of including virtually all LV remodeling patterns based on LV volume, mass index and RWT has been proposed [27, 28]. The individual patterns were related to the type of injury or overload, and some of them were closely associated with an adverse outcome, while others appeared to be adaptive or the expression of physiologic response to increased load. Most notably, the term eccentric LVH was applied to patterns with enlarged (dilated) ventricles.

The combination of increased LV mass with the development of eccentric LVH is directly related to deterioration of LV performance. It has been demonstrated that the inability of hypertrophy to keep pace with an abnormally high ventricular wall stress may be responsible for depressed LV function [29]. We hypothesized that ESE-derived parameters of LV performance might be useful in predicting the ensuing cavity enlargement and the decline in wall thickness-to-cavity radius or RWT.

In our study, the results of the ESE testing showed that LV contractility, that is the intrinsic ability of the myocardium to generate force and to shorten independently of changes in loading conditions (afterload and preload), as assessed by the evaluation of echo-derived ESPVR (LV elastance) in response to exercise, can predict subsequent development of adverse LV remodeling. The prognostic value of echo-derived ESVPR at increasing heart rate has been widely demonstrated with either ESE or pharmacological stress echo [30–32], but, to our knowledge, this study provides novel information over the relationship between impairment of contractility and the prediction of later adverse LV remodeling.

The transition from a compensated stage of hypertrophy to adverse LV remodeling could be related to the mechanisms by which the inotropic reserve decreases from the initial compensatory phase, albeit the presence of LV dysfunction, to the final stage of exhaustion and progressive deterioration [33]. This form of LVH alters supply-to-demand balance by increasing myocardial oxygen consumption and is often associated with impaired myocardial vascularisation [34], myocyte loss, unfavourable changes in the extracellular matrix composition and fibrosis [35].

The most important study limitation was the reproducibility of LV metrics. As it has been recently pointed out [36], the magnitude of changes of the echocardiographic measures is small enough to fall into the intra- and inter-observer margin of error for these measurements. However, we perform the best of our efforts to guarantee the best reproducibility of the measures by limiting the sources of errors, especially because of the collaborative acquisition and interpretation (I.F., N.R.P., and M.M) of the echocardiographic data both at rest and during exercise, that brought about the intra- and inter-observer variability coefficient for our laboratory within a reasonable range.

## Conclusion

A compromised ESE-derived peak ESPVR, that reflects impaired LV contractility, resulted to be the most powerful predictor of adverse LV remodeling, defined on the basis of LV volumes and RWT, in patients with HF with reduced or mildly reduced LVEF.

## Abbreviations

BNP: Brain Natriuretic Peptide
BP: Blood Pressure
CO: Cardiac Output
CPO: Cardiac Power Output
CPOM: Cardiac Power output-to-mass
EDV: End-Diastolic Volume
EDVi: End-Diastolic Volume index
EF: Ejection Fraction
ESE: Exercise Stress Echocardiography
ESP: End-Systolic Pressure
ESPVR: End-Systolic Pressure Volume Relation
ESV: End-Systolic Volume
ESVi: End-Systolic Volume index
HF: Heart Failure
LV: Left Ventricular
LVEF: Left Ventricular Ejection Fraction
LVH: Left Ventricular Hypertrophy
MBP: Mean Blood Pressure
NYHA: New York Heart Association
RWT: Relative Wall Thickness
